# Functional Implications of Sleep Development

**DOI:** 10.1371/journal.pbio.0030178

**Published:** 2005-05-17

**Authors:** Jerome M Siegel

## Abstract

Why do we sleep? The sleep patterns and mechanisms that occur throughout development may give us a clue.

Although frazzled new parents may beg to differ, infants do sleep more than adults. This sleep pattern is seen in a wide variety of mammalian species, with some obvious selective advantages. Sleep is a time of reduced body and brain metabolic rate [1,2], allowing energy conservation, particularly if a warm place is available, as can be provided by a compliant parent or sibling. The sleeping, quiescent infant is also less likely to attract predators and is easier to transport. At the earliest ages, infants who have not yet opened their eyes and whose cortex is not yet developed have limited learning opportunities from interactions with the outside world: another reason for reduced waking.

But sleep comes in many forms. Evolutionary arguments may make sense for slow-wave deep sleep patterns at birth that are associated with a general shutdown of the brain, but may not provide such an obvious explanation for the relative predominance of rapid eye movement (REM) sleep. For example, in human neonates REM sleep constitutes approximately eight hours per day, or 50% of the total sleep time, whereas human adults devote less than two hours per day, or 20% of their seven to eight hours of sleep time, to REM sleep [3]. REM sleep is characterized by high brain metabolic and neuronal activity rates [4], reduced muscle tone, irregular and relatively automatic respiration uncoupled from its usual regulatory mechanisms [5], and diminished thermoregulation [6]. These properties seem maladaptive, which suggests that there must be some compensatory survival benefit for REM sleep to have persisted. Could REM sleep play a particularly important role in development?

Interesting evidence for this hypothesis has come from studying the effects of REM sleep deprivation on the development of the visual system. It is known that the occlusion of one eye during the maturation of visual connections that occurs after birth causes the open eye to acquire more central connections than the closed eye. This disproportionate representation seems to result from a difference of activity in the optic nerve between the open eye and closed eye [7]. Although early ideas that REM sleep was necessary for brain plasticity might suggest that REM sleep deprivation would prevent this reorganization, just the reverse occurs. REM sleep deprivation accelerates the shift of connections to favor the open eye [8,9]. Rather than facilitating change [10], REM sleep may therefore be a source of endogenous activity that tends to prevent altered sensory stimulation from causing abnormal connections to form. REM sleep may prevent the programmed cell death and the pruning of connections that occurs when critical synapses are not stimulated.

Another possible role for neonatal REM sleep might be in thermoregulation of the growing brain. It is known that nonREM sleep tends to cool the brain, reducing its thermoregulatory set point [11]. In contrast, REM sleep tends to heat certain brain regions [12]. The nonREM–REM alternation comprises a thermoregulatory oscillation.

It is often assumed that the amount of time spent in different sleep states is determined by processes controlled by the cerebral cortex. The emphasis on the cortical role in sleep may result more from the technical ease of recording electroencephalograms from the cortex than from persuasive functional evidence. At birth, cortical metabolism and neuronal firing are minimal [13], yet this is the time of greatest sleep. In adults, damage to the cortex produces little or no change in sleep, indicating that the signal for sleep does not originate in or at least does not require the cortex [14]. Animals with proportionally larger cortices do not have more REM or nonREM sleep time than animals with relatively little cortex [15]. The effects of long-term sleep deprivation have been shown to be largely autonomic in nature, including elevated body temperature, skin lesions, and increased food intake [16]. Such effects cannot be duplicated by any cortical lesions. However, many of these symptoms appear to be consistent with hypothalamic dysfunction [17,18].

Evolutionary evidence also suggests that the cortex may be a relatively recent participant in REM sleep. Plesiomorphic (primitive) mammals such as the egg-laying echidna and platypus have large amounts of REM-sleep-like activity in brainstem structures at birth [19,20]. The brainstem is the key region for REM sleep generation, being both necessary and sufficient for its occurrence [4]. However, the cortex of these animals scarcely changes activity during these states, showing slow-wave patterns during the REM sleep state. In this respect the sleep of placental mammals may represent ontogeny recapitulating phylogeny, since a reduction in electroencephalogram power is a late-developing component of REM sleep.

A prominent feature of REM sleep is the rapid eye movements and associated twitches that define the state. These are particularly marked and vigorous in neonates. It has been shown that twitches with some resemblance to REM sleep activity are present in the isolated spinal cord of neonates and diminish in the transected cord of older animals [13]. This has suggested to some that a primal phasic activity of the central nervous system transforms postnatally over an extended time period into the very different brainstem-generated pattern seen in adults. But in this issue of *PLoS Biology*, Karlsson et al. [21] show that this is not the case. In a set of technically demanding experiments, they demonstrate a remarkable similarity between sleep control mechanisms in the one-week-old rat and those in the adult cat, and by implication throughout the mammalian line.

By severing the connections to and from the forebrain (cerebral cortex and associated structures), Karlsson et al. were able to study sleep-related activity in the midbrain and brainstem. They described the rat homologs of the medullary neurons that induce the atonia seen in sleeping adult cats and narcoleptic dogs [22,23,24] (Figure 1). More rostrally, they identified neural activity in the region of the locus coeruleus that facilitates movement and report contrasting inhibitory activity in the adjacent subcoeruleus region, again paralleling studies in the cat [25,26,27]. They also found cells that appear to generate or at least contribute to the twitches of REM sleep.

**Figure 1:**
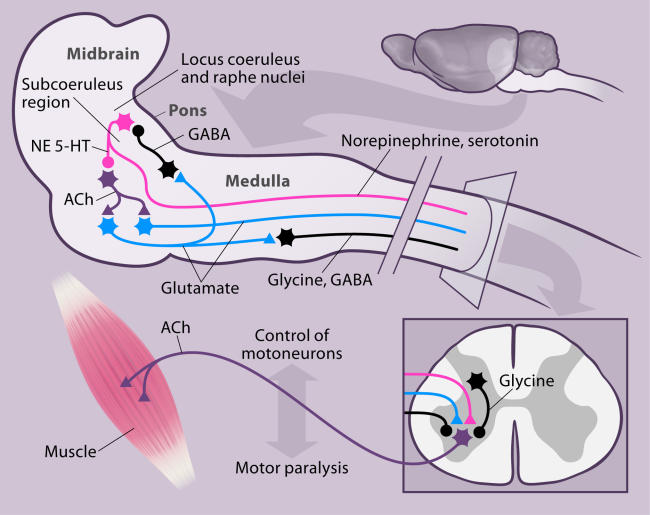
Model of Some of the Major Systems Involved in Regulating Muscle Activity in REM Sleep Drawn on a Sagittal Section of the Brainstem Cholinergic (ACh) neurons in the pons, which are under the inhibitory control of noradrenergic (NE) and serotonergic (5-HT) neurons, trigger REM sleep. They activate descending glutamatergic neurons, which in turn activate glycinergic and GABAergic neurons. Other glycinergic interneurons in the spinal cord are also activated by unknown descending inputs. The release of glycine and GABA inhibits motoneurons. The descending glutamatergic pathway also activates GABAergic interneurons, which inhibit noradrenergic and serotonergic neurons. The reduction in norepinephrine and serotonin release during REM sleep disfacilitates motoneurons. Descending glutamatergic neurons that connect directly to motoneurons produce phasic excitation during REM sleep. The net result of the action of this network is an absence of muscle tone in the “antigravity” muscles in REM sleep, interrupted by twitches (see text for references).

The similarities to the adult cat's REM sleep control mechanisms are so striking that what becomes interesting are the small differences that are reported. The locus coeruleus REM “sleep-off cells,” which are active in waking, reduce activity in nonREM sleep, and cease activity in REM sleep, appear to not have long-duration waveforms in the neonatal animals examined by Karlsson et al., unlike the case of the adult rat and cat [28,29]. Another difference is the apparent absence of the cessation of dorsal raphe (serotonin) unit discharge in REM sleep. Although the authors speculate that this is due to the absence of forebrain connections in their experimental preparation, it has been shown that forebrain mechanisms are not necessary for this cessation of raphe activity in adult cats [30]. However, identification of the narrow dorsal raphe nucleus is difficult even in adult cats, and it is certainly possible that these neurons were overlooked in the neonatal rat.

The upshot of these findings is a picture of a largely mature REM sleep generator mechanism at birth. The developmental progression of REM sleep signs, particularly the reduction in sleep duration and the development of the characteristic reduction in electroencephalogram voltage to a waking-like pattern in REM sleep, may result from the maturation of the targets of these brainstem systems, the modulation of these generator mechanisms by developing systems, or a relatively subtle maturing of connections within the REM sleep generator systems. This work pushes the probable organization of the REM sleep generator system in rats back to before one week of age, possibly to an in utero stage.

What does all this say about the function of REM sleep? Although we are left with the same initial speculations, the neonatal model provides a different perspective for approaching these functions. It is particularly useful to know that key elements of the REM sleep system are present in neonatal rats, since these animals are ideal subjects for in vitro studies of tissue slices [31,32]. It is not practical to perform in vitro experiments on the adult brainstem. However, there has always been some question as to whether studies of neonatal brainstems would be applicable to the question of adult REM sleep mechanisms. One can now imagine examining the metabolism and membrane characteristics of these critical cell groups as a means of gaining better insight into REM sleep function. However, as Karlsson et al.'s work demonstrates [21], most of the neurons of interest are not homogenously concentrated in any easily targeted region. Identifying the individual neurons of interest in vitro remains a challenge. This challenge will have to be surmounted in order to identify the control mechanism and better understand the function of REM sleep.

## Abbreviation

REM: rapid eye movement
